# Effects of electromagnetic waves on pathogenic viruses and relevant mechanisms: a review

**DOI:** 10.1186/s12985-022-01889-w

**Published:** 2022-10-12

**Authors:** Yi Xiao, Li Zhao, Ruiyun Peng

**Affiliations:** Beijing Institute of Radiation Medicine, Yard 27, Taiping Road, 100850 Beijing, P.R. China

**Keywords:** Electromagnetic wave, Pathogenic virus, Inactivation, Physical resonance, Thermal effect, Nonthermal effect

## Abstract

Pathogenic viral infections have become a serious public health issue worldwide. Viruses can infect all cell-based organisms and cause varying injuries and damage, resulting in diseases or even death. With the prevalence of highly pathogenic viruses, such as severe acute respiratory syndrome coronavirus 2 (SARS-CoV-2), it is urgent to develop efficient and safe approaches to inactivate pathogenic viruses. Traditional methods of inactivating pathogenic viruses are practical but have several limitations. Electromagnetic waves, with high penetration capacity, physical resonance, and non-contamination, have emerged as a potential strategy to inactivate pathogenic viruses and have attracted increasing attention. This paper reviews the recent literature on the effects of electromagnetic waves on pathogenic viruses and their mechanisms, as well as promising applications of electromagnetic waves to inactivate pathogenic viruses, to provide new ideas and methods for this inactivation.

## Background

Many viruses are fast-spreading, long-lasting, and highly pathogenic, with the potential to cause global pandemics and severe human health hazards. Prevention, detection, inspection, elimination, and treatment are critical steps to block viral spreading. Pathogenic viruses’ rapid and effective elimination includes preventative, protective, and source-site elimination. Inactivating pathogenic viruses by physiological destruction to reduce their infective, pathogenic, and reproductive abilities is a powerful approach to their elimination. Traditional methods, including high temperatures, chemical agents, and ionizing radiation, can effectively inactivate pathogenic viruses. However, these methods remain subject to several limitations. Therefore, there is still an urgent need to develop innovative strategies to inactivate pathogenic viruses.

Electromagnetic wave radiation has potential as a practical way to inactivate pathogenic viruses due to its high penetrating ability, rapid and homogeneous heating, resonance with microorganisms, and plasma release [1–3]. The ability of electromagnetic waves to inactivate pathogenic viruses has been demonstrated in the last century [4]. In recent years, the applications of electromagnetic waves in the inactivation of pathogenic viruses have attracted increasing attention. This paper reviews the effects of electromagnetic waves on pathogenic viruses and their mechanisms, which might provide helpful guidance for both basic and applied research.

## Effect of electromagnetic waves on pathogenic viruses

### Effect of electromagnetic waves on the morphology of pathogenic viruses

The morphological characteristics of viruses can reflect function, such as survival and infectious ability. It has been demonstrated that electromagnetic waves can disrupt the morphology of viruses, especially ultrahigh-frequency (UHF) and extremely high-frequency (EHF) electromagnetic waves.

Bacteriophage MS2 (MS2) is frequently used in a variety of research fields, such as the disinfection evaluation, kinetic modeling (aqueous), and biological characterization of viral molecules [5, 6]. Wu found that 2450 MHz, 700 W microwaves caused aggregation and significant shrinkage of water-borne MS2 phage after direct radiation for 1 min [1]. Surface rupture of the MS2 phage was also observed after further investigation [7]. Kaczmarczyk [8] exposed a coronavirus 229E (CoV-229E) sample suspension to a 95 GHz millimeter wave with a power density between 70 and 100 W/cm^2^ for 0.1 s. Large holes could be detected on the rough spherical envelope of the virus, which caused loss of their content. Electromagnetic wave exposure can be destructive to viral morphology. However, the changes in morphological properties, such as shape, diameter, and surface smoothness, after the exposure of a virus to electromagnetic radiation are not yet well understood. Therefore, it is important to analyze the relationship between the disruption of morphological features and functions, which might provide a valuable and convenient indicator for evaluating virus inactivation [1].

### Effect of electromagnetic waves on the structure of pathogenic viruses

The viral structure generally consists of the internal nucleic acid (RNA or DNA) and the external capsid. The nucleic acid determines the genetic and replication properties of the virus. The capsid, the outer layer of regularly arranged protein subunits, is the main scaffolding structure and an antigenic component of the viral particle and protects the nucleic acids. Most viruses have an outer envelope structure consisting of lipids and glycoproteins. Furthermore, envelope proteins determine receptor specificity and act as primary antigens, which the host immune system can recognize. An intact structure ensures the integrity and genetic stability of the virus.

#### Viral nucleic acids and proteins

Studies have reported that electromagnetic waves, especially UHF electromagnetic waves, can destroy the RNA of pathogenic viruses. Wu [1] directly exposed the water-borne MS2 virus to 2450 MHz microwaves for 2 min, and the encoding genes of A protein, capsid protein, replicase protein, and lysis protein was analyzed by gel electrophoresis and reverse transcription-polymerase chain reaction (RT‒PCR). These genes was destroyed gradually with increasing power density and even disappeared at the highest power density. For example, the expression of the A protein gene (934 bp) was obviously decreased after exposure to 119 and 385 W electromagnetic waves, and the expression was completely abolished when the power density was adjusted to 700 W. These data suggested that electromagnetic waves can dose-dependently damage the nucleic acid structure of a virus.

Recent studies suggested that the effect of electromagnetic waves on pathogenic viral proteins is predominantly based on their indirect thermal effects on the medium and their indirect impact on protein synthesis by disrupting nucleic acids [1, 3, 8, 9]. However, nonthermal effects could also alter the polarity or structure of viral proteins [1, 10, 11]. The direct impact of electromagnetic waves on essential structural/nonstructural proteins, such as capsid proteins, envelope proteins, or spike proteins of pathogenic viruses, still needs further investigation. Recently, it has been proposed that 2 min of 2.45 GHz, 700 W electromagnetic radiation could denature SARS-CoV-2 spike protein, which is critical for the entry of SARS-CoV-2 into host cells, through the formation of hot spots and the interaction of the oscillating electric field with different parts of protein charges via a pure electromagnetic effect [12].

#### Envelope of pathogenic viruses

The envelope of pathogenic viruses is closely associated with the infectious or pathogenic ability. Several studies have reported that UHF and super-high-frequency (SHF) electromagnetic waves can disrupt the envelope of pathogenic viruses. As described above, obvious holes could be detected on the viral envelope of coronavirus 229E after exposure to a 95 GHz millimeter wave at a power density between 70 and 100 W/cm² for 0.1 s [8]. The resonant energy transfer effect of electromagnetic waves could generate sufficient stress to disrupt the viral envelope structure. For enveloped viruses, infectivity or certain activities are usually reduced or completely lost after envelope disruption [13, 14]. Yang [13] directly exposed influenza virus H3N2 (H3N2) and influenza virus H1N1 (H1N1) to microwaves at 8.35 GHz, 320 W/m² and 7 GHz, 308 W/m², respectively, for 15 min. RT‒PCR was performed to compare the RNA signal of the electromagnetic wave-exposed pathogenic virus and a fractured model, which was subjected to several cycles of freezing in liquid nitrogen and immediate thawing. The results showed excellent agreement between the RNA signals of the two models. These results suggested that the physical structure of the virus was broken and that the envelope structure was disrupted after microwave exposure.

### Effect of electromagnetic waves on the activity of pathogenic viruses

The activity of a virus can be characterized by its ability to infect, replicate, transcribe, and so on. Viral infectivity or activity is usually evaluated by measuring viral titers using quantitative plaque assay analysis, median tissue culture infectious dose (TCID50), or luciferase reporter gene activity. However, it can also be directly evaluated by the isolation of live virus or the analysis of viral antigens, virus particles density, viral survival rate, and so on.

It has been reported that UHF, SHF, and EHF electromagnetic waves can directly inactivate viral aerosols or water-borne viruses. Wu [1] exposed MS2 phage aerosols, generated by a laboratory nebulizing device, to 2450 MHz and 700 W electromagnetic waves for 1.7 min, and the survival rate of the MS2 phage was only 8.66%. Similar to MS2 virus aerosols, 91.3% of waterborne MS2 was inactivated within 1.5 min after exposure to electromagnetic waves at the same dose. Moreover, the ability of electromagnetic radiation to inactivate MS2 virus was positively associated with power density and exposure time. However, when the inactivation efficiency reached its maximum, it could not be improved by extending the exposure time or increasing the power density. For example, the minimum survival rate of the MS2 virus was between 2.65% and 4.37% after exposure to 2450 MHz and 700 W electromagnetic waves, and no significant alteration could be detected by increasing the exposure time. Siddharta [3] radiated cell culture suspensions containing hepatitis C virus (HCV)/human immunodeficiency virus type 1 (HIV-1) with electromagnetic waves at 2450 MHz and 360 W. They found that the virus titers were significantly reduced after 3 min of exposure, indicating that electromagnetic wave radiation is effective against HCV and HIV-1 infectivity and could contribute to the prevention of virus transmission even in the context of coexposure. When low-power electromagnetic waves at 2450 MHz, 90 W, or 180 W were used to radiate HCV cell cultures and HIV-1 suspensions, there was no alteration in virus titer as determined by luciferase reporter gene activity and no significant change in virus infectivity. Even at 600 and 800 W for 1 min, there was no significant loss of infectivity for either virus, which is thought to be related to the electromagnetic wave radiation power and critical temperature action time.

Kaczmarczyk [8] first demonstrated the lethality of EHF electromagnetic waves against water-borne pathogenic viruses in 2021. They exposed coronavirus 229E or poliovirus (PV) samples to 95 GHz electromagnetic waves with power densities varying between 70 and 100 W/cm^2^ for 2 s. There two pathogenic viruses were inactivated with efficiencies of 99.98% and 99.375%, respectively, which indicated that EHF electromagnetic waves had promise in the field of virus inactivation.

The efficiency of UHF electromagnetic wave-mediated virus inactivation was also evaluated on different media, such as breast milk and some materials commonly used in life. Researchers exposed anesthesia masks contaminated with adenovirus (ADV), poliovirus type 1 (PV-1), herpes virus 1 (HV-1), and rhinovirus (RHV) to electromagnetic radiation at 2450 MHz and 720 W. They reported that ADV and PV-1 antigen detection changed to negative, and the titers of HV-1, PIV-3, and RHV decreased to zero, indicating that all of the viruses were completely inactivated after exposure for more than 4 min [15, 16]. Elhafi [17] exposed swabs contaminated with avian infectious bronchitis virus (IBV), avian pneumovirus (APV), Newcastle disease virus (NDV), and avian influenza virus (AIV) directly to 2450 MHz, 900 W microwaves for 20 s, and all of these viruses lost their infectivity. Among them, APV and IBV were further tested in tracheal organ cultures prepared from chicken embryos after five passages. Although the virus could not be isolated, viral nucleic acids were still detectable by RT‒PCR. Ben-Shoshan [18] directly exposed 15 cytomegalovirus (CMV) antigen-positive breast milk samples to electromagnetic waves at 2450 MHz and 750 W for 30 s. Antigen detection using the Shell-Vial method showed complete inactivation of CMV. However, complete inactivation was not achieved in 2 out of 15 samples at 500 W, indicating a positive relationship between the inactivation efficiency and the power of the electromagnetic waves.

It is also noteworthy that Yang [13] predicted the resonant frequency between electromagnetic waves and viruses according to the established physical model. A suspension of H3N2 virus particles with a density of 7.5 × 10^14^ m^− 3^, generated by virus-susceptible Madin Darby canine kidney (MDCK) cells, was directly exposed to electromagnetic waves at 8 GHz and 820 W/m² for 15 min. The inactivation rate of the H3N2 virus was up to 100%. However, only 38% of the H3N2 virus was inactivated at the theoretical threshold of 82 W/m^2^, which indicated that the efficiency of electromagnetic radiation-mediated inactivation of the virus was closely related to the power density. Based on this study, Barbora [14] calculated the resonant frequency range (8.5–20 GHz) between electromagnetic waves and SARS-CoV-2 and deduced that the exposure of 7.5 × 10^14^ m^− 3^ SARS-CoV-2 virus particles to electromagnetic waves with frequencies of 10–17 GHz and a power density of 14.5 ± 1 W/m^2^ for approximately 15 min would result in 100% inactivation. A recent study by Wang [19] clarified that the resonant frequencies of SARS-CoV-2 are 4 and 7.5 GHz, confirming the existence of resonant frequencies independent of virus titer.

In summary, electromagnetic waves can affect virus activity both in aerosols and in suspensions, as well as on the surfaces of objects. The inactivation efficiency was found to be closely associated with the frequency and power of the electromagnetic waves and with the medium used for virus growth. Furthermore, physical resonance-based electromagnetic frequencies are prominent in the field of virus inactivation [2, 13]. Until now, electromagnetic waves’ effects on pathogenic viruses’ activity have mainly focused on the alteration of infectious ability. Due to the complex mechanisms, few studies have reported the effects of electromagnetic waves on the replication and transcription of pathogenic viruses.

### Mechanisms underlying the inactivation of pathogenic viruses by electromagnetic waves

The mechanisms underlying the inactivation of viruses by electromagnetic waves, which are closely related to the type of virus, the frequency and power of the electromagnetic waves, and the viral growth medium, are still largely unexplored. Recent studies have mainly focused on the mechanisms of thermal, nonthermal, and structural resonance energy transfer effects.

#### Thermal effects

The thermal effect refers to the temperature increase induced by the high-speed rotation, collision, and friction of polar molecules in tissues under electromagnetic waves. Due to this property, electromagnetic waves can raise the temperature of the virus beyond the physiological tolerance threshold, resulting in viral death. However, viruses contain few polar molecules, which suggests that direct thermal effects on viruses are rare [1]. In comparison, there are many more polar molecules in the medium and surrounding environment, such as water molecules, and they move according to the alternating electric field excited by electromagnetic waves to generate heat by friction. Then, the heat is transferred to the virus to raise its temperature. When the tolerance threshold is exceeded, the nucleic acids and proteins are destroyed, ultimately decreasing infectivity or even inactivating the virus.

Several groups have reported that electromagnetic waves can reduce the infectivity of viruses through thermal effects [1, 3, 8]. Kaczmarczyk [8] exposed coronavirus 229E suspensions to electromagnetic waves at 95 GHz with a power density between 70 and 100 W/cm² for 0.2–0.7 s. The results revealed that a 100 °C increase in temperature during this process contributed to the destruction of virus morphology and a reduction in viral activity. These thermal effects might be attributed to the effects of electromagnetic waves on the water molecules around them. Siddharta [3] subjected HCV-containing cell culture suspensions with different genotypes, including GT1a, GT2a, GT3a, GT4a, GT5a, GT6a, and GT7a, to electromagnetic wave radiation at a frequency of 2450 MHz and powers of 90 W, 180 W, 360 W, 600 W, and 800 W. Electromagnetic wave radiation decreased the infectivity of the virus or completely inactivated the virus when the cell culture medium temperature increased from 26 ℃ to 92 ℃. However, when HCV was exposed to electromagnetic waves at low power (90 or 180 W for 3 min) or higher power for a short time (600 or 800 W for 1 min), no obvious elevation in the temperature occurred, and no significant alteration in virus infectivity or activity was observed.

The above results indicated that the thermal effect of electromagnetic waves is a critical factor affecting the infectivity or activity of pathogenic viruses. Moreover, numerous studies have demonstrated that the thermal effect of electromagnetic exposure produces higher inactivation efficiency on pathogenic viruses than UV-C and conventional heating [8, 20–24].

#### Nonthermal effects

In addition to thermal effects, electromagnetic waves can alter the polarity of molecules, such as microbial proteins and nucleic acids, and cause the rotation and vibration of molecules, which results in reduced viability or even death [10]. It is believed that the rapid conversion of the polarity of electromagnetic waves could cause protein polarization and lead to the twisting and bending of protein structures, ultimately resulting in protein denaturation [11].

The nonthermal effects of electromagnetic waves on the inactivation of viruses are still controversial, but most studies find positive results [1, 25]. We have described above that electromagnetic waves can directly penetrate the outer shell protein of the MS2 virus and destroy viral nucleic acids. Moreover, MS2 viral aerosols are much more sensitive to electromagnetic waves than water-borne MS2. Due to the less polar molecules, such as water molecules in the surrounding environment of MS2 viral aerosols, nonthermal effects might play pivotal roles in the electromagnetic wave-mediated inactivation of viruses [1].

#### Physical resonance properties

The resonance phenomenon refers to the tendency of a physical system to absorb more energy from its surroundings at its natural vibration frequency and wavelength. Resonance occurs in many parts of nature. Viruses are known to resonate in the confined-acoustic dipolar mode with microwaves of the same frequency, which is a resonance phenomenon [2, 13, 26]. The resonance mode of electromagnetic wave-virus interactions has attracted increasing attention. The efficient structure-resonant energy transfer (SRET) effect from electromagnetic waves to confined acoustic vibrations (CAVs) in viruses could result in the fracture of the viral membrane through opposite core-shell oscillations. Furthermore, the overall SRET efficiency is related to the properties of the surrounding environment, among which the size of the virus particle and pH determine the resonant frequency and energy absorption, respectively [2, 13, 19].

The physical resonance effect of electromagnetic waves has played a pivotal role in the inactivation of enveloped viruses, which are surrounded by a bilayer membrane embedded with viral proteins. Researchers found that the inactivation of H3N2 by electromagnetic waves with a frequency of 6 GHz and a power density of 486 W/m² was mainly caused by the physical rupture of the envelope through the resonance effect [13]. The temperature of the H3N2 suspension increased only 7 °C after exposure for 15 min; however, the inactivation of the human H3N2 virus by thermal heating requires a temperature above 55 °C [9]. A similar phenomenon was also observed for viruses such as SARS-CoV-2 and H3N1 [13, 14]. Moreover, the inactivation of viruses by electromagnetic waves did not cause degradation of the viral RNA genome [1, 13, 14]. Therefore, physical resonance but not thermal effects contributed to the inactivation of the H3N2 virus [13].

Compared to the thermal effect of electromagnetic waves, the inactivation of viruses by physical resonance requires lower dose parameters, below the microwave safety standards set by the Institute of Electrical and Electronics Engineers (IEEE) [2, 13]. The resonant frequency and power dose were determined by the physical characteristics of the virus, such as the particle size and elasticity, and all of the viruses located in the resonant frequency could be efficiently targeted and inactivated. Due to the high penetration, lack of ionizing radiation, and good safety, the nonthermal SRET effect-mediated inactivation of viruses is promising in the treatment of human malignant diseases caused by pathogenic viruses [14, 26].

## Discussion

Electromagnetic waves are highly effective against virus aerosols based on the realization of virus inactivation in the liquid phase and on the surfaces of different media [1, 26], which is a breakthrough and has great significance for the control of virus transmission and prevention of social epidemics. In addition, the discovery of the physical resonance properties of electromagnetic waves is of great significance in the field. As long as the resonant frequency between a certain viral body and electromagnetic waves is known, all viruses within the resonant frequency range of the wound can be targeted, which is not possible with traditional virus inactivation techniques [13, 14, 26]. The electromagnetic inactivation of viruses is a prospective study with great value and potential for research and application.

Compared with traditional virus elimination technology, electromagnetic waves show the simplicity, high efficiency, practicality, and environmental friendliness of killing viruses with their own unique physical characteristics [2, 13]. However, there are still many challenges. First, the current knowledge is limited to the physical properties of electromagnetic waves, while the mechanisms of energy utilization during electromagnetic wave radiation have not been revealed [10, 27]. Microwaves, including millimeter waves, have been widely used to study virus inactivation and its mechanisms; however, investigations of electromagnetic waves at other frequencies, especially frequencies ranging from 100 kHz to 300 MHz and 300 GHz to 10 THz, have not been reported. Second, the mechanisms underlying the electromagnetic wave-mediated killing of pathogenic viruses have not been well defined, and SRET has been investigated only on spherical and rod-shaped viruses [2]. In addition, the characteristics of viruses, such as the small size of virus particles, absence of a cellular structure, easy mutation, and rapid propagation, could prevent the inactivation of viruses. Electromagnetic wave technologies still need to be improved to overcome the obstacles to the inactivation of pathogenic viruses. Finally, the high absorption of radiation energy by polar molecules in the medium, such as water molecules, causes energy waste. Moreover, the efficiency of SRET could be affected by several undefined mechanisms in viruses [28]. The SRET effect might also alter the viruses to adapt to the environment, causing resistance to electromagnetic waves [29].

In the future, the technologies of electromagnetic wave-mediated inactivation of viruses need further improvement. Basic science research should aim to clarify the mechanisms underlying the electromagnetic wave-mediated inactivation of viruses. For example, the mechanisms of energy utilization during the exposure of viruses to electromagnetic waves, the detailed mechanisms of nonthermal effects in the killing of pathogenic viruses, and the mechanisms of the SRET effect between electromagnetic waves and different types of viruses should be systematically clarified. Studies on application should focus on how to prevent the excessive absorption of radiation energy by polar molecules, explore the effects of electromagnetic waves at different frequencies on the various pathogenic viruses, and investigate the nonthermal effect of electromagnetic waves in the killing of pathogenic viruses.

Electromagnetic waves have emerged as a promising approach for the inactivation of pathogenic viruses. Electromagnetic wave technology could overcome the limitations of traditional antivirus technology due to its excellent advantages, including low contamination, low cost, and high efficiency in inactivating pathogenic viruses. However, further investigations should be conducted to define the parameters of electromagnetic wave technology and to clarify the mechanisms of viral inactivation.

## Conclusion

Electromagnetic wave radiation at a specific dose can destroy the structure and activity of multiple pathogenic viruses. The efficiency of viral inactivation is closely associated with the frequency, power density, and exposure time. Moreover, the underlying mechanisms include thermal effects, nonthermal effects, and structural resonance energy transfer effects. Compared with traditional antiviral technologies, electromagnetic wave-based viral inactivation has several advantages, such as simplicity, high efficiency, and low pollution. Therefore, electromagnetic wave-mediated viral inactivation has emerged as a promising antiviral technology for future application.

## Data Availability

Not applicable.

## Abbreviations

SARS-CoV-2: Severe acute respiratory syndrome coronavirus 2.
UHF: Ultrahigh frequency.
EHF: Extremely high frequency.
MS: Bacteriophage MS2.
CoV-229E: Coronavirus 229E.
RT‒PCR: Reverse transcription–polymerase chain reaction.
SHF: Superhigh frequency.
H3N2: Influenza virus H3N2.
H1N1: Influenza virus H1N1.
TCID50: Median tissue culture infectious dose.
HCV: Hepatitis C virus.
HIV-1: Human immunodeficiency virus type 1.
PV: Poliovirus.
MDCK: Madin Darby canine kidney.
ADV: Adenovirus.
PV-1: Poliovirus type 1.
HV-1: Herpes virus 1.
RHV: rhinovirus.
IBV: Infectious bronchitis virus.
APV: Avian pneumovirus.
NDV: Newcastle disease virus.
AIV: Avian influenza virus.
CVM: Cytomegalovirus.
SRE: Structure-resonant energy transfer.
CAVs: Confined acoustic vibrations.
IEEE: Institute of Electrical and Electronics Engineers.
